# Design of the Balance@Work project: systematic development, evaluation and implementation of an occupational health guideline aimed at the prevention of weight gain among employees

**DOI:** 10.1186/1471-2458-9-461

**Published:** 2009-12-14

**Authors:** Lisanne M Verweij, Karin I Proper, Andre NH Weel, Carel TJ Hulshof, Willem van Mechelen

**Affiliations:** 1 Department of Public and Occupational Health, The EMGO Institute for Health and Care Research, VU University Medical Center, Amsterdam, The Netherlands; 2 Body@Work, Research Center Physical Activity, Work and Health, TNO-VUmc, Amsterdam, The Netherlands; 3 Netherlands Society of Occupational Medicine, Utrecht, The Netherlands; 4 Coronel Institute of Occupational Health, Academic Medical Center (AMC), Amsterdam, The Netherlands

## Abstract

**Background:**

Occupational health professionals may play an important role in preventive health promotion activities for employees. However, due to a lack of knowledge and evidence- and practice based methods and strategies, interventions are hardly being implemented by occupational physicians to date. The aim of the Balance@Work project is to develop, evaluate, and implement an occupational health guideline aimed at the prevention of weight gain among employees.

**Methods:**

Following the guideline development protocol of the Netherlands Society of Occupational Medicine and the Intervention Mapping protocol, the guideline was developed based on literature, interviews with relevant stakeholders, and consensus among an expert group. The guideline consists of an individual and an environmental component. The individual component includes recommendations for occupational physicians on how to promote physical activity and healthy dietary behavior based on principles of motivational interviewing. The environmental component contains an obesogenic environment assessment tool. The guideline is evaluated in a randomised controlled trial among 20 occupational physicians. Occupational physicians in the intervention group apply the guideline to eligible workers during 6 months. Occupational physicians in the control group provide care as usual. Measurements take place at baseline and 6, 12, and 18 months thereafter. Primary outcome measures include waist circumference, daily physical activity and dietary behavior. Secondary outcome measures include sedentary behavior, determinants of behavior change, body weight and body mass index, cardiovascular disease risk profile, and quality of life. Additionally, productivity, absenteeism, and cost-effectiveness are assessed.

**Discussion:**

Improving workers' daily physical activity and dietary behavior may prevent weight gain and subsequently improve workers' health, increase productivity, and reduce absenteeism. After an effect- and process evaluation the guideline will be adjusted and, after authorisation, published. Together with several implementation aids, the published guideline will be disseminated broadly by the Netherlands Society of Occupational Medicine.

**Trial Registration:**

ISRCTN73545254/NTR1190

## Background

Overweight is one of the world's most challenging public health problems [1]. The prevalence of overweight has reached epidemic proportions in most Western countries, including the Netherlands. Currently, over 30% of the Dutch working population is overweight and around 6% is obese [2]. The prevalence of overweight is expected to increase substantially over the next years [3]. The average gain in weight is calculated to vary between 0.4-0.9 kg per year [4,5]. This trend is a cause for concern as overweight has been associated with an increased risk for type 2 diabetes, cardiovascular diseases, and some cancers, as well as with higher absenteeism rates and lower productivity levels [6-9]. Consequently, the overweight-related mortality, direct costs (health care costs) and indirect costs (costs of sick leave, loss of production) may increase [10,11].

Despite worldwide awareness of the obesity epidemic, tackling this public health problem remains difficult [12]. Individual treatment of overweight subjects has proven difficult because the large behavioral changes necessary for weight loss are difficult to achieve. Moreover, body weight is often regained on the long term [13,14]. Population-based prevention of weight gain, aimed at personal and environmental factors, may prove to be more efficient.

Overweight is the result of a gradual gain in weight over time as energy intake (i.e. dietary behavior) exceeds energy expenditure (i.e. physical activity). In order to prevent weight gain, a small change of 100 kcal/day is assumed necessary, which may be walking 2000 steps more per day or replacing an energy dense snack by a healthier snack [15]. This may best be achieved by inducing small behavior changes because these are more feasible to achieve and maintain [16].

The workplace is considered an appropriate setting for lifestyle promotion, as a major part of the adult population can be reached [2]. In the Netherlands, employees can be reached through health risk appraisals conducted by occupational physicians (OP). This voluntary health risk appraisal, which may consist of anthropometric measurements (such as body composition, aerobic fitness and blood values) and a subsequent advice by the OP, makes both research and intervention feasible among Dutch employees. An extensive literature study concluded that primary prevention lifestyle interventions are effective and feasible within the Dutch occupational health setting [17]. Nevertheless, OPs hardly implement weight gain prevention interventions due to a lack of knowledge and evidence-based methods and strategies. To date, activities of OPs are predominantly aimed at reducing absenteeism. Moreover, of the few OPs who do implement preventive interventions, the strategies vary and are not based on the current evidence. Based on the literature study and additional interviews, it was concluded that a need exists among OPs how to promote physical activity and dietary behavior in an effective way.

The Netherlands Society of Occupational Medicine recognises the need for improvement of the professional quality of the OPs and thus emphasises the need for a clinical practice guideline. Practice guidelines are systematically developed statements, designed to assist physicians with decisions about appropriate health care for specific clinical circumstances [18]. Practice guidelines consist of 1) a guideline text, which is formulated as a structured stepwise sequence of recommendations for practice, 2) a one page summary leaflet and 3) a background document, in which the levels of evidence for the recommendations are indicated and reference to the most important literature is given. The systematic development, evaluation and implementation of a weight gain prevention practice guideline in a careful and well-designed process is thus innovative and of major relevance. In order to perform a proper cost-, effect- and process evaluation of the guideline, the evaluation of the guideline is conducted in a randomised controlled trial. The Balance@Work project consists of three phases: phase 1. development, phase 2. evaluation, and phase 3. implementation of an occupational health guideline aimed at the prevention of weight gain among employees. This paper describes the development of an occupational health guideline, the design of the evaluation and the design of the implementation.

## Methods

### Phase 1. Development of the weight gain prevention guideline

**Figure 1:**
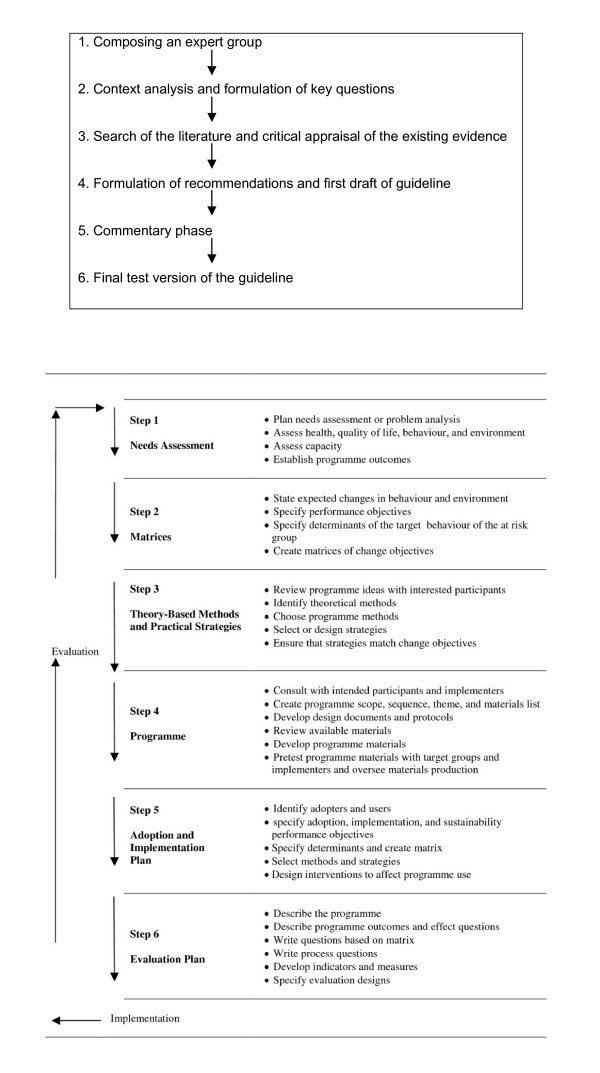
**1a Guideline development protocol of the Netherlands Society of Occupational Medicine **[19]. 1b - Intervention mapping protocol [20].

For the systematic development of the weight gain prevention guideline and associated intervention, the practice guideline development protocol of the Netherlands Society of Occupational Medicine was applied in combination with the Intervention Mapping (IM) protocol [19,20] (Figure 1a and 1b). Both protocols facilitate a stepwise process for theory- and evidence based development of guidelines and health promotion interventions respectively. In phase 1 we describe our approach to each step applied to the prevention of weight gain among employees, focusing particularly on steps 1-4 of the IM protocol. As all steps of the Netherlands Society of Occupational Medicine protocol complement those of the IM protocol, these steps were incorporated in phase 1.

#### Step 1. Needs assessment

The aim of the needs assessment was to identify the overall program objective, i.e. what is the overall program meant to accomplish [20]. The needs assessment includes a structured analysis of behavioral risk factors and environmental conditions that contribute to the health problem (i.e. weight gain among workers) via a combination of methods, such as a literature review and interviews with relevant stakeholders (i.e. OPs, employees and employers). Additionally, steps 1-3 of the Netherlands Society of Occupational Medicine protocol were added as these steps fit well with the needs assessment.

The literature search was conducted in PubMed using keywords regarding our participants (i.e. worker, employee, adult, workplace, occupational physician), intervention (i.e. controlled trial, physical activity, nutrition, health promotion, obesity prevention) and outcome measures (i.e. physical activity, nutrition, body weight). Additionally, individual studies, reviews of scientific literature and (inter)national reports on obesity and preventing weight gain were obtained. Identified risk behaviors and environmental factors were discussed in focus group interviews among seven OPs and 33 employees, and face-to-face interviews among three employers, in order to gain further in-depth knowledge of each stakeholders' needs regarding the occupational health guideline and the associated intervention. The interviews were conducted using a predefined semi-structured protocol and were digitally recorded. Following steps 1-3 of the Netherlands Society of Occupational Medicine protocol, an expert group was formed consisting of four OPs and four experts in the field of lifestyle. Involving decision makers from the start helps to move the guideline and associated intervention towards practice-based evidence, rather than just classical evidence-based practice [21]. The expert group met plenary five times during one year to discuss the context and key questions, the literature, and the existing evidence.

The needs assessment led to the identification that personal and environmental factors on both sides of the energy balance attribute to weight gain. On the energy expenditure side, a lack of physical activity as a part of daily routines was particularly identified as a risk behavior to cause weight gain [22]. Among daily physical activities, different domains can be distinguished: 1) transport-related activities, 2) work-related activities, and 3) leisure time activities and sport participation [23]. Additionally, sedentary behavior (characterized by an energy expenditure less than 2 MET, with 1 MET equivalent to the consumption of 3,5 ml O_2 _· kg^-1 ^· min^-1^), across all domains of physical activity was identified as a risk behavior for weight gain [24]. Regarding energy intake, a diet high in fat or carbohydrates and low in fiber, frequent snacking and frequent consumption of sugar-containing soft drinks were identified as risk behaviors which relate most to weight gain [1]. Finally, the influence of the 'obesogenic environment', i.e. an environment that discourages physical activity and encourages over consumption, was identified as a major risk factor for weight gain [15].

Across literature, a variety of high risk groups for weight gain were identified, such as: young starters, shift workers, blue collar workers as well as sedentary workers, those with a low social-economic status but also women with a high social-economic status, those with high job stress, those with care for children, those who stop smoking and postmenopausal women [4,25-32]. But as gradual excessive weight gain occurs across all these groups, the target group for the weight gain prevention guideline and associated intervention was specified as: workers with unhealthy physical activity and/or dietary behavior and/or workers who are overweight, according to (inter)national standards. Additionally, in order to accomplish environmental changes that facilitate behavioral changes among workers, employers were defined as a second target group.

From the needs assessment the following overall program objective emerged: to stimulate daily physical activity and healthy dietary behavior in order to prevent weight gain among Dutch workers with unhealthy behavior and/or weight, by means of a practice guideline for OPs targeting personal and environmental factors.

#### Step 2. Performance and change objectives

The second step of IM further specified the expected changes as a result of the intervention in four program objectives. Next, performance objectives were specified for each of the four program objectives (what workers are supposed to do as a result of the program), from which the most important and changeable behavioral and environmental determinants were selected [20].

The first task was to translate the selected personal risk behaviors of the needs assessment into health-promoting behavior, based on existing literature and results from the interviews. Two program objectives for increasing physical activity and two program objectives for increasing healthy dietary behavior were identified (table 1). With respect to physical activity behavior, increasing physical activity across all domains (i.e. transport-related, work-related, leisure time and sports) was encouraged. Moreover, due to the fact that sitting time during work increased over the last decades and that sedentary behavior is regarded as an independent risk factor of adverse health [33], decreasing sedentary behavior was specified as an objective. Regarding nutrition, the consumption of fruit was promoted, and a decrease in consumption of snacks was stimulated. The consumption of soft drinks was not targeted as only small observational studies found an association between soft drinks and weight gain in adults [34,35].

**Table 1:** Program objectives of the Balance@Work project.

1. Employees increase their levels of physical activity
2. Employees decrease their levels of sedentary behavior
3. Employees increase their consumption of fruit
4. Employees reduce their energy intake derived from snacks

The next step was to specify performance objectives for each of the program objectives. These constitute the specific behavioral outcomes of the program expected from the target group. Specification of the performance objectives was done via a combination of methods, namely a review of the literature, interviews with relevant stakeholders and a review of theoretical models such as Goal setting theory [36], Implementation intentions [37], the Theory of Planned Behavior [38], the Precaution Adoption Process Model [39], and the EnRG framework [40]. Based on the self-regulation theory, eight performance objectives were specified for each program objective [41]. The performance objectives for the first program objective are illustrated in table 2.

**Table 2:** Performance objectives for individual and environmental changes related to increasing the level of physical activity (program objective 1)

Individual changes related to program objective 1.
1. Employees monitor their level of physical activity
2. Employees indicate reasons to be physically active
3. Employees indicate barriers for being active
4. Employees identify solutions to take away barriers to being physically active
5. Employees decide to become more physically active
6. Employees make plans to become more physically active
7. Employees increase their physical activity
8. Employees evaluate whether the causes of insufficient activity are taken away, evaluate the effects and maintain their level of PA by enhancing their routine and preventing relapse
Environmental changes related to program objective 1.
1. OPs monitor the level of physical activity of employees
2. OPs* monitor current health policy
3. OP and employers have a positive attitude towards increasing physical activity
4. OPs* identify environmental risk factors for inactivity
5. OPs* indicate barriers for providing opportunities for employees to increase the level of physical activity
6. OPs* identify solutions to take away barriers to being physically active
7. OPs* make plans to provide opportunities for employees to increase the level of physical activity
8. OPs* provide opportunities for employees to increase the level of physical activity
9. OPs* evaluate whether the causes of insufficient activity are taken away, evaluate the effects and maintain opportunities for employees to increase the level of physical activity by company health policy and attention for relapse prevention.

* in collaboration with the employer

**Figure 2:**
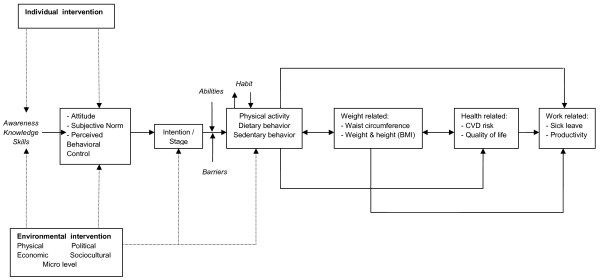
**Balance@Work Model**. Legend Figure 2 - Describing the Balance@Work intervention, aimed at improving physical activity and dietary behavior of employees in order to prevent weight gain.

The question remains how workers can be stimulated to improve their daily physical activity and dietary behavior and thus prevent weight gain. Changeable personal and environmental determinants were therefore selected that may facilitate change. This task again involved the application of information from literature, interviews, behavioral determinant theories, behavioral change theories and social ecological models. To illustrate our conceptual model, figure 2 describes the Balance@Work model, which is based on the NHF-NRG intervention and evaluation model [42].

##### Personal determinants

Based on the Theory of Planned Behavior (TPB), attitude, subjective norm and perceived behavioral control were identified as relevant behavioral determinants towards being more physically active across all domains of physical activity, and towards the consumption of fruit and low energy dense products [43,44]. The TPB specifies that these determinants on their turn influence the intention to adopt a certain behavior. A positive intention however does not always lead to an actual change in behavior (the 'intention-behavior gap'), especially in complex, habitual behaviors like daily physical activity and dietary behavior [45]. Because the presence of strong habits may limit the effect of intention as a predictor of behavior, habit strength was identified as relevant determinant [46]. Finally, knowledge, awareness and skills were found relevant in earlier weight gain prevention studies [42,43].

Determinants of behavior change were identified based on the Precaution Adoption Process Model (PAP-M). The PAP-M assumes that people behave qualitatively different at different stages of change [39]. Although the validity of behavioral change theory may be discussed, evidence exists that interventions that have applied the stages of change concept are more effective (at the short term) than non-stage matched interventions due to the distinction between motivation and action [44,47]. The stages are therefore incorporated in the Balance@Work model.

##### Environmental determinants

Several studies found an effect of the environment on the identified energy-balance related behaviors [40,48]. Relevant environmental determinants of energy-balance related behaviors were previously identified as: availability (for example bike sheds and a variety of healthy foods), management commitment and support (is health incorporated in the company's policy and does the company have a workers' representative council?), social support from co-workers, company policies (such as catering policies) and costs (food pricing, subsidised transport) [16,43]. Additionally, to overcome the intention-behavior gap, environmental opportunities should be facilitated by decision makers in order to create an environment that enables workers to change energy-balance related behaviors. Thus, environmental change interventions can best be aimed at these decision makers [44].

Finally, a matrix was developed of performance objectives crossed with the determinants, resulting in specific learn and change objectives (Additional File 1).

#### Step 3. Selecting theory-based methods and practical strategies

This step involves the selection of theory-based methods and practical strategies from literature that should lead to the desired changes. A theoretical method is a general technique or process for influencing change in personal and environmental determinants [20]. Thus, theory-based methods should improve workers' abilities and the environmental opportunities to effectively act on their motivations (i.e. overcome the intention-behavior gap). Behavioral change methods selected from the vast amount of literature were; tailored feedback, personalized risk, self-monitoring, decisional balance, re-evaluation of beliefs, reinforcement, goal setting, active learning, rewarding, mobilizing social support, skills training, and environmental changes [20,43,49]. Methods were then translated into practical strategies in order to accomplish a successful shift from motivation to action and maintenance. In translating methods into strategies, the theoretical conditions must be considered that apply to the method used [50]. For example, risk feedback may only be effective for increasing awareness when certain conditions are met, such as including both personal risk feedback (compared to a standard) and normative feedback (compared to a reference group).

Central in this self-regulation process is the formation of action goals, pursuing these goals and overcoming barriers [44]. This can be discussed during the workers' health surveillance counseling session. Because OPs stated that employees often fail to comply with health advice and express resistance to change, a more client-centered counseling technique was selected for OP based on motivational interviewing. Motivational interviewing has proven feasible and effective in primary prevention interventions targeting energy-balance related behaviors [51,52]. It aims to elicit a person's ambivalence, motivation and possibilities for change. Workers are encouraged to express thoughts, feelings and ambivalence in order to reach a decisional balance. They are then asked to decide what they may want to change, and specify implementation intentions to reach goals. Because OPs stated to have limited time, behavioral change counseling was applied. Behavioral change counseling is an adapted form of motivational interviewing, suitable for brief consultations in healthcare settings [53]. During counseling, the OP is asked to 1. provide direct advice only when asked and 2. address resistance to change, as resolving resistance is the first step to change [54].

Previous weight gain prevention research suggests that in people not yet motivated to prevent weight gain, the focus during counseling should be on awareness and attitude by means of self-monitoring, personalization of risk, tailored feedback, discussing decisional balance and beliefs. Among those who are motivated to prevent weight gain, attention should be aimed at increasing the perceived behavioral control in overcoming barriers [55] and at improving skills and stimulating social support. Additionally, formulating implementation intentions help to change habits. In order to maintain healthy behavior, rewarding and positive feedback have proven to be effective [56]. Moreover, tailored advice, use of a minimal intervention and using several contact moments showed promising results. An overview of determinants, theoretical methods and practical strategies are described in Table 3.

**Table 3:** Personal and environmental determinants, theoretical methods, practical strategies and tools identified for increasing the level of physical activity among employees (program objective 1).

Determinant	Theoretical method	Practical strategy	Tools
Knowledge	Tailored feedback	Provide verbal tailored feedback on national recommendations, benefits and possibilities	Stage matched feedback during counseling. Information in print materials and in diary.

Awareness	Personalized risk Self-monitoring	Provide verbal personal and normative feedback Facilitate and stimulate written monitoring of own behavior	Stage matched feedback during counseling. Risk communication chart. Balance@Work toolkit consisting of: a. Waist circumference measuring tape with a coloring scheme that indicates a healthy waist circumference. b. Pedometer. c. Diary containing information and logs to keep track of nutrition, physical activity and steps per day. d. Print materials on physical activity and nutrition.

Attitude	Decisional balance Re-evaluation of beliefs	Discuss the decisional balance Provide positive feedback on changes in attitude and discuss irrational thoughts	Stage matched feedback during counseling. Agenda setting chart in diary. Scales assessing importance, confidence and willingness to change. Decision matrix in diary.

Perceived behavioral control	Reinforcement Goal setting	Increase confidence by providing positive feedback and discuss difficult situations Invite to formulate implementation intentions	Stage matched feedback during counseling. Evaluation of realistic short and long term goals, reason for goals, barriers and solutions in diary.

Habit	Active learning Goal setting Rewarding	Formulate action and coping plans Invite to formulate implementation intentions Invite to formulate rewards	Stage matched feedback during counseling. Evaluation of realistic short and long term goals, reason for goals, barriers and solutions, and rewards for reached goals in diary.

Skills	Active learning Reinforcement	Encourage to formulate implementation intentions and train relapse prevention skills Provide positive feedback on skills	Stage matched feedback during counseling. Evaluation of current skills and needed skills.

Subjective norm	Mobilizing social support Skills building to reduce social pressure	Invite to formulate who can provide support Encourage to train relapse prevention skills	Stage matched feedback during counseling. Evaluation of who can provide social support in diary.

Availability	Environmental changes	Encourage to formulate environmental changes that facilitate a healthy work environment	Information in print materials. Environment scan consisting of: a checklist which provides insight in opportunities to create a healthier work environment. Examples of items are: are bike sheds and change facilities present? is the cafeteria adapted to stimulate healthy nutrition? is a rewarding system present? Feasibility and barriers for changing the environment can be scored together with the employer, as well as a start and evaluation date.
	
Management commitment and support	Mobilizing management support Rewarding	Encourage management participation Provide incentives	
	
Social support	Mobilizing social support from collegues	Encourage participation of collegues	
	
Company policy and costs	Mobilizing management to provide policy that encourages a healthy work environment	Encourage management and the workers' representative council to facilitate changes	

#### Step 4: Design of the guideline and intervention program

**Figure 3:**
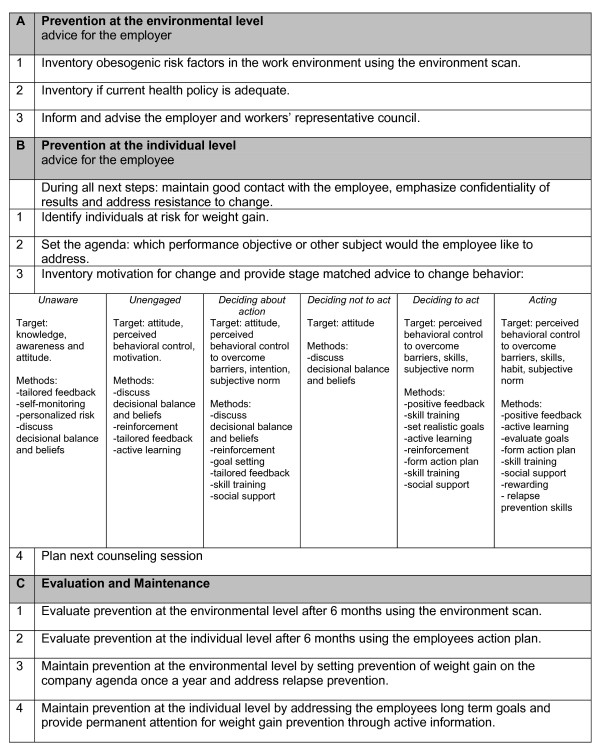
Occupational health guideline.

Step four describes the scope and sequence of the intervention and requires translation of the methods and strategies into intervention materials (table 3). The first draft of the guideline was subjected to commentary of five independent experts in the field of lifestyle. This resulted in a final test version of the guideline (step 4-6 of the Netherlands Society of Occupational Medicine protocol). Based on a critical appraisal of the evidence, experiences from the interviews, practical and ethical considerations, and consensus among the expert group, a practice guideline for OPs was developed. For the entire guideline and intervention, preventing weight gain was re-formulated as promoting physical activity and a healthy diet, in order to set a positive tone. The guideline consists of three sections: a. prevention at the environmental level (advice for the employer); b. prevention at the individual level (advice for the employee); and c. evaluation and maintenance (figure 3). The starting point for OPs to stimulate a healthy lifestyle could either be section a or b. Eventually, both sections should be addressed and re-evaluated in a cyclic manner.

With regard to prevention at the environmental level (*section a*), an environment scan was developed. This checklist helps the OP to discuss opportunities for a healthier work environment with the employer. The checklist contains previously identified determinants of weight gain, categorized for the selected physical activity behaviors (transport-related behavior, work-related activities and leisure-time activities including sport participation such as the availability of bike sheds, food policies and a company discount for the gym) and dietary behaviors (snacks and fruit consumption, for example healthy choices in vending machines and free fruit at work). By checking what the company already offers, the environment scan also shows which opportunities are lacking. For the selected determinants, feasibility and barriers for implementation can be noted. Based on this overview, environmental goals can be prioritised. To stimulate the implementation of short and long term goals, a start and evaluation date can be set with the employer. Based on the environment scan an advice for improving the obesogenic environment can be generated by the OP for the employer and the workers' representative council.

With regard to prevention at the individual level (*section b*), a stepwise minimal intervention strategy was made to facilitate the OPs individual counseling based on behavioral change counseling. As employees expressed their concern towards participating in a program provided by the OP in focus group interviews (due to expected consequences from the employer as a result of poor health), the guideline explicitly states that OPs are to maintain good contact with employees, emphasize confidentiality of results and resolve resistance to change. During the counselling sessions, first the agenda is addressed (which performance indicator or other subject would the employee like to target). Next, motivation for change is assessed and the OP provides stage matched advice to change behavior. OPs are provided with several tools derived from motivational interviewing to discuss ambivalence and increase motivation; a) an agenda setting chart, containing all target behaviors from which employees can choose what to discuss, b) a risk communication chart containing graphic information on risks for diabetes, hypertension and heart disease for different weight levels c) a chart with scales of willingness, importance and confidence to change behavior and d) a decision matrix containing pros and cons of the current behavior and target behavior. Additionally, employees are provided with a toolkit containing a waist circumference measure tape, a pedometer, existing information flyers and a diary in which target behavior can be monitored. During the counselling sessions, the OP moves the employee towards a decision balance and increases perceived behavioral control by formulating a maximum of three implementation intentions. Barriers can be discussed for each goal and the employee can set rewards for reached goals. Additionally, in order to identify environmental opportunities for reaching goals, the work environment is discussed as well as possibilities for social support.

In total, 5 counselling sessions are planned. During the first session, motivation for change is addressed. After 3 weeks a second session takes place in which goals are evaluated and updated. To enhance motivation and adherence to the personal goal behaviors, a third and fourth session are planned after 6 and 12 weeks. Due to time issues these two sessions can be by phone rather than face-to-face. Again, goals are evaluated and eventually updated. At the end of the intervention period after 6 months, a final counselling session will focus on the goals that have or have not been achieved, and on maintenance of healthy behavior.

Ultimately, the aim of the guideline is to incorporate attention for weight gain prevention in the company's health policy, which is supported by all relevant stakeholders (evaluation and maintenance, *section c*). Interviews among employers and employees revealed that an evaluation and/or maintenance plan for health initiatives are rarely exist. This section was therefore addressed separately in the guideline. Regarding prevention at the environmental level, the guideline recommends to evaluate to what extent the health policy is carried out after 6 months. In case the health policy is not or (only) partly implemented, possible reasons and barriers should be discussed. In order to maintain attention for the health policy, this issue should be addressed at least once a year in a meeting with the employer and the workers' representative council. Additionally, ongoing efforts should be made to evaluate risk factors that may induce relapse.

With respect to prevention at the individual level, short term goals should be evaluated after 12 weeks. When goals are reached, the OP is asked to compliment the employee on this achievement and to discuss long-term goals for maintaining this healthy behavior. Goals that have not been reached are discussed and the action plan is revised. Additionally, an active provision of information should be realized in order to maintain attention for healthy behavior, for example during other consultations than preventive medical examinations.

According to the commentary phase of the Netherlands Society of Occupational Medicine protocol, the draft guideline was sent to a sample of experts in the field of lifestyle. Comments, mainly concerning clarification of text and terminology, were discussed and the text was adapted.

### Phase 2. Evaluation of the weight gain prevention guideline

The guideline is currently evaluated in a randomised controlled trial among 20 OPs including approximately 600 employees. Phase 2 describes the design of this trial, population, sample size and outcome measures, as well as a plan for adoption, implementation and evaluation (Step 5 and 6 of the IM protocol).

#### Study design

OPs were randomly assigned to the intervention or control condition by an independent researcher using Random Allocation Software (version 1.0, Isfahan University of Medical Sciences, Iran). Randomization of OPs took place before the baseline measurement, as the first counseling session of the intervention group took place directly after the baseline measurement. OPs in the intervention group were trained during two days in the guideline and associated intervention, including behavioral change counselling by a professional trainer. OPs in the control group were asked to provide care as usual. OPs were asked not to reveal their condition to participating workers. The study protocol was approved by the Ethics Committee of the VU University Medical Center.

#### Study population

OPs were recruited through a direct mailing by the Netherlands Society of Occupational Medicine to their member registry. OPs providing services to one or more companies of medium to large size (> 100 workers) and conducting preventive medical examinations were eligible to participate. OPs invited employees to participate via their usual channels (written letter, intranet, face-to-face in consultation hours). Inclusion criteria were: 1. unhealthy physical activity and/or dietary behavior and/or overweight, 2. currently not being on sick leave for 21 days or longer, 3. able to complete a Dutch questionnaire and 4. having signed an informed consent form. Unhealthy physical activity was defined as workers who do not meet physical activity guidelines [57]. Unhealthy dietary behavior was defined as workers who do not meet the Dutch national dietary guidelines for fruit- and vegetable consumption and fat [58]. Overweight and obesity were defined by a waist circumference larger than 94 cm and 102 cm respectively for men and larger than 80 cm and 88 cm respectively for women [59]. Obese workers could additionally be referred to the Dutch guideline for treatment of Obesity [60]. Workers were excluded when pregnant or in case a disease or condition was present which made physical activity impossible.

#### Sample size

**Figure 4:**
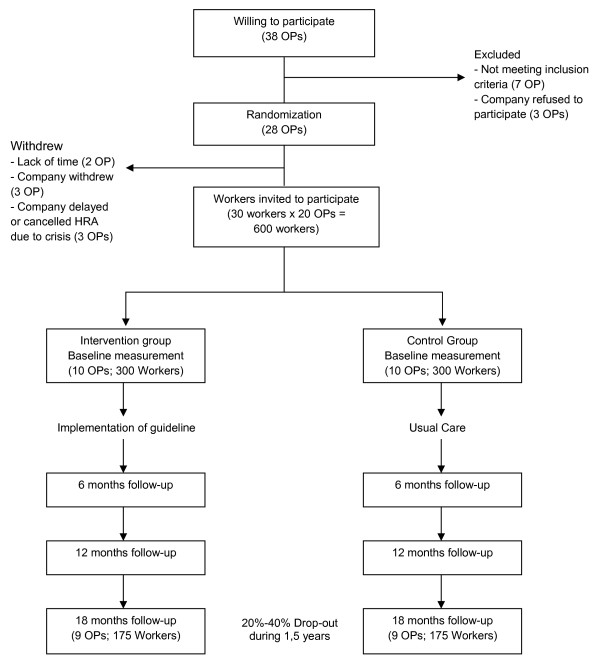
Flow chart of the study population.

The sample size was calculated according to the number of cases needed to identify an effect on waist circumference. Waist circumference was chosen over BMI as it is a more sensitive indicator of abdominal fat mass [61]. A previous study indicated that a difference in waist circumference of 1,5 cm between the intervention and control condition may be clinically relevant [43]. In an intention-to-treat analysis, 175 workers per condition are needed to detect a relative difference of 1,5 cm (standard deviation 4,5 cm) in waist circumference with a power of 80% and an alpha of 5%. As workers are clustered within OPs we adjusted for dependence of measurements with an intraclass correlation of 0.20. Taking a loss to follow-up of 20-40% into account among workers and OPs until the final measurement, the initial study population should consist of approximately 300 workers among 10 OPs in each study group (figure 4).

#### Measurements

Questionnaires are sent to employees prior to the initial health risk appraisal. Questionnaires are collected by OPs or their assistants during the health risk appraisal. Biomedical measurements are performed by trained OPs or their assistants following a standard protocol. Follow-up measurements take place after 6, 12 and 18 months.

#### Primary outcome measures

##### Waist circumference

Waist circumference is measured as midway between the lower rib margin and the iliac crest to the nearest 0.1 cm [59,62]. Participants are measured in standing position without heavy outer garments and with emptied pockets, breathing out gently. To standardize waist circumference measurement, OPs or assistants were provided with a Seca 201 waist circumference measure (Seca, Hamburg, Germany). Additionally, waist circumference is measured in a random sample of workers (10-20%) at each measurement by an independent researcher.

##### Physical activity

Daily physical activity is measured with The Short Questionnaire to Assess Health Enhancing Physical Activity (SQUASH). The SQUASH assesses activities across 4 domains: 1) commuting activities (walking and cycling to/from work), 2) physical activity at work, 3) household activities and 4) leisure time activities (walking, cycling, gardening, chores and sports). Participants are asked to report the number of days per week spent on each activity during an average week in the past month, as well as the number of minutes per day and the intensity of each activity. The SQUASH is a fairly reliable (spearman's correlation coefficient = 0.58) and reasonably valid (spearman's correlation coefficient = 0.45) questionnaire [63]. Total levels of daily physical activity are assessed by two items on the number of days per week moderate intensity activities are performed (such as walking and cycling) for at least 30 minutes (this can be built up by blocks of 10 minutes) and how often per week vigorous intensity leisure time activities ("which make you sweat") are performed during 20 minutes. These items relate to international physical activity guidelines [57] as well as to the Dutch guidelines [64].

##### Dietary behaviour

Dietary behavior is operationalized as the intake of fruit, vegetables, fruit juice and snacks. These items were selected from the validated Short Fruit and Vegetable questionnaire (validity r = 0.50) [65] and the Fat list (validity r = 0.70) [66]. The number of days per week and the number of daily servings of fruit, vegetables and fruit juice is measured with five items on citrus fruit, other fruits, cooked vegetables, raw vegetables and fruit juice. The consumption of energy dense snacks is assessed by the number of days per week and the number of servings per day in 7 categories (peanuts, chips, cakes, candy bars, biscuits, other cookies and fries). Cut-off points for each behavior are adapted from recommendations of the National Nutrition Centre (The Hague, The Netherlands).

#### Secondary outcome measures

##### Sedentary behaviour

*S*edentary behavior is specified for work and for leisure time. Sedentary behavior at work is asked as the number of minutes per day spent on computer use, meetings and other activities. Leisure time sedentary behavior is asked as the number of minutes per day spent on watching tv, computer use and other activities. Answers are specified for week days and weekend days [67]. Additionally, total duration of sedentary behavior is asked per week as well as per weekend day during the last 7 days (IPAQ).

##### Determinants of behavior change

Based on the TPB, attitude, subjective norm, perceived behavioral control and intention are included on a 5-point Likert scale for physical activity, dietary behavior and watching one's weight [55]. Additionally, 3 items on barriers and 4 items on habit strength are added [46]. Motivational stage of change, based on PAP-M, is assessed for cycling to work, physical activity at work, leisure time physical activity, fruit consumption, snack consumption and watching one's weight [39].

##### Weight-related factors

Body weight and body height are measured with the participants standing without shoes and heavy outer garments. Weight is measured to the nearest 0,5 kg. Height is measured to the nearest 0,1 cm. Participants are asked to push their heels softly to the wall, or the back of stadiometer [62]. BMI is calculated from measured height and weight as kg/m^2^.

##### Health-related factors

Systolic and diastolic blood pressure (in mmHG) are measured in a seated position, after several minutes of rest. OPs are asked to follow the standard Dutch protocol for blood pressure measurements [68]. Total serum cholesterol is measured, and if feasible for OPs, HDL and LDL cholesterol are measured as well (mmol/l). As OPs apply the guideline during their daily practice, the type of instruments used for blood pressure and cholesterol measurements may vary between OPs. The type of instruments used is asked retrospectively, and OPs are requested to use the same instruments throughout the trial.

Cardiovascular disease risk profile is calculated using the European Systematic Coronary Risk Evaluation instrument (SCORE) based on systolic blood pressure (mmHG), cholesterol levels (mmol/l) gender, age and smoking status [69]. Quality of life is measured by the validated EuroQol [70]. Presence of chronic disease and medication use are asked for hypercholesterolemia, hypertension, angina, heart disease, myocardial infarct, COPD, arthritis, cancer, depression and diabetes.

##### Work related factors

Productivity and absenteeism are measured by the HPQ. In the HPQ, work productivity is conceptualized as a measure of actual performance in relation to possible performance (i.e. presenteeism) [71]. Productivity is assessed with two items, measuring absolute and differential productivity (i.e., productivity compared with others). Absenteeism is measured as the absolute number of workdays missed in the past 4 weeks because of problems with physical or mental health. The HPQ shows good concordance with employer records of work absence and work productivity [72,73].

#### Process evaluation

The process evaluation will assess eight aspects after the intervention period at 6 months by questionnaire and focus group interviews: context (organizational characteristics that affect the intervention), recruitment (sources and procedures used to recruit OPs and employees), reach (attendance rates of employees), dose delivered (the amount of intervention components actually delivered by the OP), dose received (the extent to which employees use materials or components recommended by program), participant's attitudes (the OPs and employees attitudes toward the quality of the intervention in terms of credibility and usefulness), fidelity (the extent to which the intervention was delivered as planned), and the link between results and process (to explain possible success or failures of the intervention) [74,75]. Moreover, OPs and workers are asked to rate the intervention with an overall score on a scale 1-10. Regarding fidelity, the extent to which OPs adhere to the guideline is assessed with performance indicators. Additionally, the OPs competence in behavior change counselling is assessed by rating three taped counselling sessions with the behavior change counselling index [76]. From each tape 10 minutes are randomly selected and scored by the MI trainer, who then provides feedback to the OPs. The behavior change counselling index shows acceptable levels of validity, reliability and responsiveness [76].

#### Economic evaluation

An economic evaluation will be conducted alongside the trial and include a cost-effectiveness analysis and a cost-utility analysis. Both analyses will be performed from a societal perspective and a company perspective. The time horizon will be 18 months, similar to the trial. The analysis will be performed according to the intention-to-treat principle. From the societal perspective, in the cost-effectiveness analysis the difference in incremental costs (in €) between the conditions will be related to the difference in waist circumference (in % change) between the intervention and control group. The costs include intervention costs (costs that are directly related to the intervention, such as the costs of training OPs and costs of intervention materials), direct health care costs (for example, costs of physiotherapy and hospitalisation) and indirect health care costs (such as costs for a healthy lifestyle, such as sports contribution fees and sports clothing). Costs will be valued using Dutch guideline prices for economic evaluation. With respect to the company perspective, the costs of the intervention and other costs related to the company will be included (such as reduced productivity and absenteeism) and compared to the difference in waist circumference (in cm) between the conditions. Absenteeism and productivity are measured every 3 months with the HPQ using cost-diaries [73].

The cost-utility analysis will focus on the difference in costs (in €) between the conditions per QALY (Quality Adjusted Life Year) gained. Utilities will be measured using the social tariff of EuroQol with Dutch reference values. Bootstrapping will be used for pair-wise comparison of the mean differences in costs between the two groups. Confidence intervals (95%) will be obtained by bias corrected and accelerated bootstrapping. Cost-effectiveness and cost-utility ratios will be calculated by dividing the difference in the mean costs between the study groups by the difference in the mean effects. Acceptability curves will be calculated, showing the probability that the guideline is cost-effective at a specific ratio. Furthermore, sensitivity analyses will be performed to assess the robustness of the results. All analyses will be performed using SPSS 15.0 (SPSS Inc. Chicago, Illinois, USA).

#### Data analysis

The effectiveness of the weight gain prevention guideline will be analyzed after 6 months (short term) and after 18 months (long term) by means of multilevel analyses. Multilevel analyses accounts for the clustering of observations of workers within the same OP, as well as repeated measurements within one worker. Due to randomization at the OP level, short term data will be analyzed with two levels: 1. worker and 2. OP. Long term data will be analyzed with three levels: 1. time (3 follow-up measurements at 6, 12 and 18 months), 2. worker and 3. OP. Both crude and adjusted analyses will be performed. The multilevel analyses using the follow-up measurement (i.e. 6 months) as dependent variable will be adjusted possible confounders such as, gender, age and education. These variables will also be checked for effect modification. The long-term effect of the intervention will be analyzed using all three follow-up measurements (i.e. 6, 12 and 18 months) adjusted for possible confounders. Effect modification is checked. All statistical analyses will be performed according to an intention-to-treat principle. The investigator analysing the data is blinded to the randomization. A two tailed significance level of p < 0.05 is considered statistically significant. Analyses will be performed with SPSS 15.0 (SPSS Inc. Chicago, Illinois, USA) and MLwiN for multilevel statistical analyses.

### Phase 3. Implementation of the weight gain prevention guideline

Based on the (cost-) effectiveness evaluation and process evaluation, the guideline will be adapted. At this stage, the expert group will be consulted for advice on finalising and implementing the definite guideline in OPs in the Netherlands. Additionally, the Diffusion of Innovation Theory is used, which describes the decision-making process which occur through a series of communication channels over a period of time among the members of a similar social system [77]. The final guideline will consist of 1) the guideline text, which is formulated as a structured stepwise sequence of recommendations for practice, 2) a one page summary leaflet and 3) a background document, in which the levels of evidence for the recommendations are indicated and reference to the most important literature is given. Additionally, implementation tools will be produced, such as knowledge tests, checklists, desktop summaries, and employer and employee versions of the guideline. After authorisation by the Netherlands Society of Occupational Medicine, the guideline will be published and disseminated among OPs and occupational health services across the Netherlands. Training in application of the guideline will be organised. Additionally, the guideline may be incorporated in post-graduate and refresher courses and in the primary OP-training at the schools of occupational medicine. The control group will receive the training after the follow up period of the trial is terminated.

## Discussion

The purpose of this paper was to outline the rationale and development of a new occupational health guideline aimed at preventing weight gain. With a focus on both physical activity and dietary behavior, using individual counselling and considering environmental influences, this guideline may have great potential. Practice guidelines are particularly useful if there is a large variation in current practice; if they contain new evidence with an important impact on health management; if they affect many individuals at high risk or involve such high costs that even small changes in practice could have major impact on health outcomes or resources [18]. An occupational health guideline aimed at the prevention of weight gain may therefore be important because 1) there is a need to address overweight on a larger scale in the Netherlands, as overweight is associated with an enormous public health impact as well an economic burden, 2) it enhances the professional quality of OPs and 3) it provides OPs with a practical guideline on how to advice on preventing weight gain.

Several limitations must be mentioned in this study design. First, as the guideline consisted of several components it may not be possible to evaluate the separate effect of each guideline component. Second, not all parameters could be standardized, such as the recruitment of workers which is done in a variety of ways by different OPs. This, however, reflects the practice-based nature of guidelines and may also be a strength of this study, as each OP provides tailored feedback [21]. Finally, the two day training may be too short as the behavioral change counselling technique is new to most OPs.

This study has several strong points as well. To our knowledge, this is the first study to extensively evaluate a practice guideline aimed at prevention. Moreover, after evaluation the guideline will be implemented broadly in the Netherlands. Also, as the guideline is developed together with relevant stakeholders, most of the intervention components can be implemented at larger scale without concerns. Finally, participating occupational physicians are distributed throughout the Netherlands in different types of companies, resulting in a heterogeneous employee sample.

The systematic development of the guideline according to the evidence-based guideline development protocol of the Netherlands Society of Occupational Medicine, in combination with the IM protocol is an innovative element in combining evidence- and practice based medicine. Based on a (cost) effectiveness and process evaluation, the guideline can be adapted before implementation on a larger scale in OPs throughout the Netherlands.

## Competing interests

The author(s) declare that they have no competing interests.

## Authors' contributions

LV designed the intervention protocol and wrote the manuscript. KP wrote the initial study protocol and was involved in preparations for the study. KP, CH, AW and WM provided intellectual input and had a role in supervision. All authors have read and approved the final version of the manuscript.

## Pre-publication history

The pre-publication history for this paper can be accessed here:

http://www.biomedcentral.com/1471-2458/9/461/prepub

## Supplementary Material

Additional file 1 Performance objectives related to changes in individual determinants with regard to increasing the level of physical activity of employees (program objective 1). Click here for file
