# Supplementary material for: Design of the Balance@Work project: systematic development, evaluation and implementation of an occupational health guideline aimed at the prevention of weight gain among employees
Source: BMC Public Health. 2009 Dec 14;9:461. doi: 10.1186/1471-2458-9-461 (PMC2799413; doi:10.1186/1471-2458-9-461)
Supplement: Additional file 1 — Performance objectives related to changes in individual determinants with regard to increasing the level of physical activity of employees (program objective 1). [file 1471-2458-9-461-S1.DOC]

**Additional File 1.** Performance objectives related to changes in individual determinants with regard to increasing the level of physical activity of employees (program objective 1).

| **Performance objectives** | **Individual determinants** | | | | | |
| --- | --- | --- | --- | --- | --- | --- |
|  | Knowledge | Awareness | Attitude | Skills | Perceived behavioral control | Subjective Norm |
| 1. Employees monitor their level of physical activity.  2. Employees indicate reasons to be physically active  3. Employees indicate barriers for being active  4. Employees identify solutions to take away barriers to being physically active  5. Employees decide to become more physically active    6. Employees make plans to become more physically active  7. Employees increase their physical activity  8. Employees evaluate whether the causes of insufficient activity are taken away, evaluate the effects and maintain their level of PA by enhancing their routine and preventing relapse | Employees know the PA recommendations  Employees know about the health benefits of being physically active  Employees know about their barriers for being physically active  Employees learn how to identify difficult situations and can indicate personal solutions  Employees know how to become more physically active  Employees know how to increase their physical activity  Employees know how to evaluate and maintain their physical activity | Employees become aware of their own PA levels  Employees monitor and report the their own PA  Employees become aware of the personal relevance of being physically active  Employees become aware of the personal relevance of being physically active  Employees become aware of barriers that prevent them from being physically active  Employees become aware of what to change to become more physically active  Employees become aware of what to do to become more physically active  Employees are aware of the relevance to evaluate and maintain their physical activity | Employees feel positive about being physically active  Employees feel positive about overcoming barriers with their solutions  Employees feel positive about becoming more physically active  Employees feel positive about their plan for being more physically active.  Employees feel positive about increasing their physical activity  Employees feel positive about evaluating and maintaining their physical activity | Employees learn how to compare their PA to these recommendations  Employees can describe their reasons for being physically active  Employees can describe their barriers for being active  Employees learn to compare and describe solutions to barriers  Employees learn how to be more physically active  Employees learn to set feasible goals (characterized by small behavioural changes)  Employees can increase their physical activity  Employees can evaluate behavioral changes, indicate causes for success or failure and maintain their physical activity | Employees feel confident about  registering their daily PA  Employees feel confident about being physically active  Employees feel confident about overcoming barriers to being physically active  Employees feel confident about being more physically active  Employees feel confident about being able to act according to the formulated implementation intentions, especially in difficult situations  Employees feel confident about increasing their physical activity  Employees feel confident about evaluating and maintaining their physical activity | Employees indicate that being physically active with colleagues, friends and family can be stimulating  Employees are aware that colleagues, friends and family can help overcome barriers to being physically active  Employees make colleagues, friends and family aware to help them become more physically active  Employees engage colleagues, friends and family to help them become more physically active  Employees increasing their physical activity with help of colleagues, friends and family |
